# Examining the Factor Structure of a Risk Assessment Inventory in Young Offenders: FER-R, Risk and Resource Assessment Form

**DOI:** 10.3390/ijerph19020756

**Published:** 2022-01-11

**Authors:** Paula Alarcón, Ricardo Pérez-Luco, Sergio Chesta, Lorena Wenger, Andrés Concha-Salgado, Eduardo García-Cueto

**Affiliations:** 1 Departamento de Psicología, Universidad de La Frontera, Temuco 4811322, Chile; paula.alarcon@ufrontera.cl (P.A.); sergio.chesta@ufrontera.cl (S.C.); lorena.wenger@ufrontera.cl (L.W.); andres.concha@ufrontera.cl (A.C.-S.); 2 Departamento de Psicología, Universidad de Oviedo, 33003 Oviedo, Spain; cueto@uniovi.es

**Keywords:** young offenders, risk assessment, protective factors, factorial validity, juvenile delinquency

## Abstract

The FER-R, Risk and Resource Assessment Form, is a multidimensional inventory of structured professional judgment that assesses criminogenic risks and resources for the design and management of individualized intervention plans with criminally sanctioned adolescents. The aim of this study was to examine the psychometric properties of the FER-R, reviewing its factorial structure to contribute evidence of convergent and discriminant construct validity in a sample of adolescents sentenced for crimes in Chile. For each domain (risks and resources) with its respective facets, a unidimensional bifactor structure (CFA-BF) was obtained, with adequate indices of fit that confirmed its construct validity, while the convergent validity was demonstrated with the YLS/CMI and the divergent validity with two MACI scales. The FER-R adds factorial validity to the evidence of the previously reported predictive validity, making it a robust inventory for the evaluation of young offenders, and a relevant tool to manage differentiated interventions in Chile, with a high potential for use in Latin America. The importance of finding a suitable balance in assessing risks and protective factors is discussed, in order to manage interventions adjusted to the needs of the adolescents to promote their criminal desistance.

## 1. Introduction

The study of social behavior during adolescence, and especially adolescent-offender evaluation, is a special challenge; it offers a window of opportunity to enhance their development and psychosocial well-being [1] and, likewise, allows us to recognize multiple variables involved in the onset and persistence of antisocial behaviors in this phase of the life cycle [2].

The increase in evidence-based practices and the modernization of justice systems in different countries, and recently in Ibero-America, has led to the incorporation of risk-assessment tools to support judicial decision-making and the management of intervention programs.

Risk-assessment tools have evolved from the first and second generation, focused almost exclusively on the prediction of recidivism, towards tools that, on the one hand integrate structured professional judgment, and on the other, concentrate on intervention management (third and fourth generation). For this, the central objectives in the measurements are the dynamic factors, susceptible to change, conceptualized as the youth’s criminogenic needs, with these being the key focal points of the interventions.

Risk-assessment tools have diversified and offer important advantages over traditional clinical evaluations; they serve to establish evidence-based practices both in specialized programs for offenders (violent, sexual, juvenile offenders) and in community interventions. These instruments have been developed mainly in North America, Europe, and Australia, and only in recent years in Latin America [3,4].

Evidence in delinquency-risk assessment derives mostly from the risk–need–responsivity (RNR) model, which over the last 30 years has allowed evaluation of criminogenic factors in a structured manner, defining criminogenic risks, as well as those factors that increase the likelihood of beginning or persisting a criminal trajectory [5,6].

The systematic reviews and meta-analyses of the assessment instruments of criminogenic risks, defined as factors that increase the probability of continuing to offend, have reported their predictive validity through the estimation of the area under curve (AUC). The prediction ranges reported by the most frequently used instruments for young people vary from AUC = 0.65 to AUC = 0.83, and today there is an ample supply of these tools published in texts and practical guides, but with little development in the Latin American region [5,6,7,8,9].

The use of risk-assessment tools has not matched psychometric studies of cross-cultural validity or factorial invariance analysis, that not only add evidence of predictive validity in recidivism, but also demonstrate that the structure of central risk factors described in the RNR model is replicated [5].

In a recent study, Mei et al. [10] reviewed the factorial structures of risk-assessment tools in specific populations presenting geographic and cultural differences. Since assessment factors—when examined in other populations—require empirical support to confirm their existence, the study focused on the behavior of dynamic criminogenic factors [11]. This is particularly important in juvenile delinquency, where intervention management is recognized as a central objective, since it is required that the resources and interests that will ultimately act as protective factors in their life trajectory be assessed also [12,13,14].

In this light, the most recent evidence in risk-assessment tools becomes highly consistent when demonstrating the need to balance the measurement between criminogenic risks and protective factors, especially in young offenders. Kleeven, de Vries Robbé, and Mulder [15], and Barnes-Lee [16] highlight that evaluating young people’s strengths and resources not only improves the predictive capacity of non-recidivism, but it establishes what is crucial about the protective factors to guide the management of the interventions and promote desistance.

Based on the evidence, two ideas should be highlighted: (i) that assessment tools should consider both criminogenic and protective factors to increase the effectiveness of interventions with young offenders, and (ii) it is necessary to advance in the study of the factorial validity of risk-assessment tools in different sociocultural contexts, as well as, the need to develop local risk-assessment scales that are more sensitive to specific groups.

The Youth Level of Service/Case Management Inventory (YLS/CMI) [17] and the Structured Assessment of Violence Risk in Youth (SAVRY) [18] are the most reported instruments in their application to young offenders, both derivatives of the RNR model.

The YLS/CMI is one of the instruments with the most international evidence. The predictive validity reported in meta-analyses is an AUC between 0.54 and 0.75, with a moderate effect size for overall recidivism, of r = 0.28 [6,19]. Follow-up of the YLS/CMI in Spain, through adaptation of the IGI-J [20,21], shows ability to discriminate between recidivists and non-recidivists. Recently, the high predictive capacity of the YLS/CMI has been observed, with AUC values ranging from 0.79 to 0.83 [22]. In Latin America, studies are limited, with Brazil [23] and Chile being the only exceptions. In the latter, there are two preliminary adaptations: (i) Fundación Paz Ciudadana [24] reports low internal consistency indices (from 0.36 to 0.67 in the subscales and 0.86 for the total) and three of the eight scales do not significantly discriminate between recidivists and non-recidivists; (ii) Chesta and Alarcón [25] report satisfactory levels in internal consistency for the total scale (alpha = 0.90), but in six subscales report an alpha below 0.70. The scales education/employment, and leisure/recreation do not discriminate between offenders and non-offenders, coinciding with a study in Brazil [23]. The YLS/CMI indicators in Latin America are still insufficient.

In the Hispanic population, a high degree of concordance has been reported in YLS/CMI and SAVRY measurements for predicting recidivism [13,26]; however, the SAVRY also measures protective factors, and these show a negative relationship with all YLS/CMI factors [13].

Viljoen et al. [14] indicate that the SAVRY measures protective factors, but its measurement is more sensitive to recognizing their absence than their variability in predicting non-recidivism.

In summary, in the Latin American context, there is little development and application of risk-assessment instruments in adolescents, and if they are applied, they do not have an exhaustive psychometric study, with follow-up and verification of evidence of validity to give robustness to the measurements and guide the management and decision-making of the intervention [3].

Based on the previous background, it is imperative to study the risk-assessment tools used with Latin American adolescents, particularly in Chile, and to consider the conditions described in the evidence as a priority, integrating criminogenic risk factors and protective factors to manage intervention programs with young offenders, as well as to support with robust psychometric evidence the scales that are most sensitive to the needs of local adolescents. Responding to these needs, the present study shows empirical evidence of the validity of a structured professional judgment inventory, developed and studied in Chile.

The *Risk and Resource Assessment Form*, FER-R (its Spanish acronym, from “*Ficha de Evaluación de Riesgos y Recursos*”), is a rationally constructed inventory based on evidence from the RNR model and developmental criminology, together with direct supervisory experience of professional teams intervening with Chilean young offenders since 2002 [27]. It consists of 8 factors and 57 items distributed in 2 domains: Risks and Resources. The first factor (interventions) assesses static risk (non-modifiable), the next five (education, peers, family, drugs, and attitudes) assess dynamic risks, susceptible to change; and the last two (protective resources and youth’s interests) assess personal and contextual adaptative resources that act as protective factors. The FER-R has a 24-month follow-up study of 100 adolescents [28], which shows overall predictive validity in 68.3% of cases and the effect size for predicting recidivism is robust (AUC = 0.73), with a 95% confidence interval (0.63–0.83). It also has an inter-rater reliability study [29] by Intra-class Correlation Coefficient analysis, obtaining a CCI value = 0.858 and reliability by internal consistency with satisfactory indices for risks (alpha 0.70–0.98) and for resources (alpha 0.80–0.94).

These results show an adequate and robust predictive utility of the FER-R for criminal recidivism, and if the magnitude of the partial prediction is considered, three dynamic criminogenic risk factors of greater prediction are highlighted: (1) education (AUC = 0.74), and specifically progressive school disengagement (AUC = 0.78); (2) peers with high criminal commitment (AUC = 0.71); and (3) family (AUC = 0.66), in particular the weakness of parental supervision (AUC = 0.66); drugs and negative attitudes have a lower predictive weight (AUC = 0.63). In resources, similar indices are obtained. The youth’s interests possess good predictive capacity (AUC = 0.67) of non-recidivism (desistance). Finally, the FER-R has a characterization study [30] with 486 adolescents, which demonstrates the capacity of the inventory to discriminate according to sex (male and female), type of delinquency exhibited (persistent or transitory), and criminal recidivism detected two years after the assessment.

The FER-R has also been used to evaluate the impact of family interventions in treatment programs for adolescents with delinquent behavior [31]. It adds a greater number of items to the family risk factor, which is very relevant as it delves into an area with distinctive cultural particularities of the Latin American context that may affect adolescents differently. All of the above add evidence on the impact that this inventory can have for the design of pertinent intervention programs.

The general objective of this study was to examine the psychometric properties of the FER-R in young offenders in Chile, and the specific objectives were: (a) to examine its factorial validity, (b) to examine convergent, divergent, and discriminant validity, (c) to determine reliability, and (d) to comparatively characterize the presence of risks and resources in the participants of the sample studied.

## 2. Materials and Methods

### 2.1. Participants

The study population was young people convicted under the Adolescent Criminal Responsibility Law in Chile, enrolled in intervention programs in four regions of the central-south of the country between 2008 and 2012 (The data correspond to two different studies financed by the Chilean National Council for Science and Technology -CONICYT, currently ANID-, FONDECYT project Nº 1070397 [32], and FONDEF project D08i-1205 [33]). The adolescent population in Chile for the 15–19 age cohort in 2010 was estimated to be 1,426,634, and 11,256 were convicted between 2008 and 2012 [34]. A total of 649 adolescent response protocols were selected by non-probabilistic convenience sampling in two research stages; in the first stage, 263 valid protocols were obtained from male adolescents in 2008, and in the second stage, 386 protocols were obtained from male (326) and female (60) adolescents between 2011 and 2012 distributed according to type of sanction in the four study regions (see Table 1). The time period in which the data were collected includes two generations of adolescents. However, both groups of study participants were equivalent in age (t = −0.11; *p* = 0.913) and schooling (t = −1.25; *p* = 0.212) and their proportional distributions did not differ in rural or urban origin (χ^2^ = 3.015; *p* = 0.221) and Mapuche or non-Mapuche origin (χ^2^ = 0.090; *p* = 0.765). All of them met the inclusion criteria: (a) criminal record in judicial files; (b) being serving a sanction, and; (c) voluntariness in their participation, manifested by signing an informed consent form.

### 2.2. Design

Two sub-samples were taken from the total sample with complete protocols for the different analyses: 633 for factorial validity and internal consistency, and 442 for convergent, divergent, and discriminant validity, using an instrumental design [35] for the final resolution of the study.

### 2.3. Instruments

*Risk and Resource Assessment Form*, *FER-R*, the acronym in Spanish for “*Ficha de Evaluación de Riesgos y Recursos*”, is an inventory of structured professional judgment that measures criminogenic risks, static and dynamic, along with adaptation resources in young offenders. It was developed in Chile by Paula Alarcón [27] and has previously published evidence of predictive reliability and validity [28]. It consists of 57 items, 39 that measure criminogenic risks (0–39 points), and 18 that measure adaptation resources (0–18 points). The scores on the FER-R are expressed in eight areas: (F1) *impact of previous interventions* (convictions); (F2) *education*, which includes the domains F2a, *school disengagement*, and F2b, *behavior problems*; (F3) *relationship with peers*; (F4) *family*, which includes the domains F4a, *weak supervision*, F4b, *approval of criminal behavior*, and F4c, *abuse*; (F5) *drugs*; (F6) *clear attitudes or tendencies*; (F7) *personal and family protective resources*; and (F8) *the youth’s interests*. The FER-R is scored by presence (1) or absence (0) and has a structured coding guide [36].

**Figure 1:**
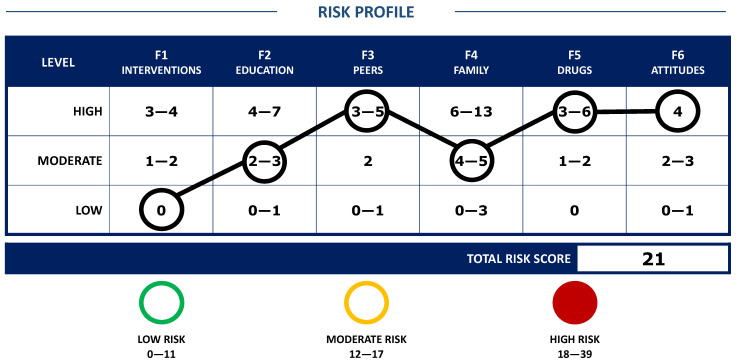
Example for presentation of results in the FER-R risk profile.

In the risks domain, the scores are standardized as low, moderate, and high in each facet, creating a risk profile that can identify the criminogenic needs of greatest urgency for the intervention (see Figure 1); and the total risk index that varies between 0 and 39 is estimated with cut-off points categorized as low-risk, <11, moderate-risk, 12–17, and high-risk >18, values derived from a two-year follow-up study [28].

**Figure 2:**
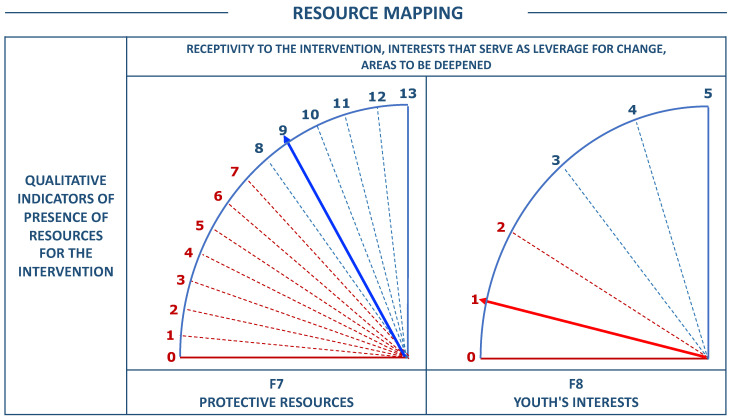
Example for mapping of FER-R resources.

In the resources domain, the score is also standardized, but independently for each facet. The graph generated for protective resources goes from 0 to 13 points, with a cut-off score of 8, and the graph for the youth’s interests from 0 to 5 points, with a cut-off score of 3 (see Figure 2). This allows a differentiated consideration in the design of interventions, using interests for the activation of protective resources and risk reduction [37].

The *YLS*/*CMI* is also an inventory of structured professional judgment, derived from a tool used in the adult judicial system, the LSI-R, which has vast evidence in different countries to evaluate criminogenic risks [17]. It consists of 42 items and is divided into eight subscales: previous and current convictions, family situation and parental role, education and employment, relationships with peers, substance abuse, free-time use, personality and behavior, and attitudes/tendencies. Each item is scored as present or absent, giving a total score between 0 and 42 points. According to this total score, the adolescents are categorized into four recidivism risk levels: low, moderate, high, or very high. The YLS/CMI [17] has an adaptation in Chile derived from its French-Canadian version, the IRNC [38], and a version revised and adapted through a preliminary validity study in Chile, YLS/CMI [25].

The *MACI*, the Millon Adolescent Clinical Inventory [39], is a rationally constructed, clinical self-report instrument to assess personality patterns and difficulties inherent in adolescence. It is comprised of 160 true–false items that make up a total of 31 scales, grouped into 12 *personality patterns*, 8 *expressed concerns*, and 7 *clinical syndromes*. In addition, it has three control scales and one validity scale. Its psychometric properties are described by its author for adolescents between 13 and 19 years. In Chile, it is a widely studied instrument and has a version adapted to Chilean standards [40].

### 2.4. Procedure

The assessment of the FER-R and YLS/CMI inventories was carried out by the professionals responsible for each case within the intervention programs, responding simultaneously to both inventories, for which each professional received a minimum of 16 h of direct training in risk-assessment methodology and in the assessment guides of each instrument, and were subsequently supervised by the researchers to review their scores and sources of information (at least three). The MACI was applied by the same professionals, but was answered directly by the adolescents through self-report in the intervention context.

### 2.5. Ethical Safeguards

The data acquisition adhered to the fundamental principles of bioethics (autonomy, beneficence, non-maleficence, and justice), and was reviewed ethically by the Chilean National Council for Science and Technology (CONICYT), which awarded and funded the projects in a national public competition. The implemented ethical protocol involved, prior to the data collection, managing the authorizations of the entity responsible for the execution of penal sanctions of adolescents in Chile, and also of the leadership of the organizations participating in the study and in charge of the direct intervention with young people. The University signed formal research collaboration agreements with each of them (The three national public institutions responsible for juvenile justice in Chile: National Service for Children, SENAME, Ministry of Justice, MINJU and Undersecretary for Crime Prevention, SPD; and two collaborating State agencies responsible for the implementation of sanctions in the study regions: Land of Hope Foundation, FTDE and Children’s Defense Council, CODENI). Given that the assessments were always performed by the professionals responsible for the intervention in each case, each was asked to sign a confidentiality agreement. The evaluated adolescents were invited to participate in the study within the framework of the sentence they were serving, and signed a consent form in which they agreed to participate in the study. Finally, an anonymized database was consolidated that was safeguarded, ensuring confidentiality for the analysis under the responsibility of the principal investigators.

### 2.6. Data Analysis

For the examination of the internal structure of the FER-R, two confirmatory factor analyses were performed with a bifactor model (CFA-BF) using the MPLUS program version 7.3 [41] and the method of mean- and variance-adjusted weighted least squares (WLSMV) with the polychoric correlation matrix [42]. The following were used to evaluate the fit of the model: comparative fit index (CFI), Tucker–Lewis index (TLI), and root mean square error of approximation (RMSEA). An adequate fit of the model considered CFI and TLI values greater than 0.90 and RMSEA values equal to or less than 0.08 [43,44]. Once the factorial structure had been determined, the reliability was estimated for each domain.

To obtain evidence of convergent validity of the FER-R, the nonparametric Spearman’s Rho correlation with the YLS/CMI was estimated [25]; for divergent validity, the total risk score of the FER-R was correlated with the same test with two personality-pattern scales of the MACI Inventory in its version adapted in Chile, (e4) dramatization and (e5) egocentrism, which measure general characteristics of most adolescents without constituting a construct related to criminogenic risks [40]. For discriminant validity, the sample was divided into recidivists and non-recidivists with a retrospective criterion, using as a measure the presence of previous offenses (with and without antecedents) and estimating mean differences (Student’s *t*) and effect size (Cohen’s d) for the contrast between both groups. These analyses were performed using SPSS 23 software (IBM, Chile, License UFRO) and the online calculator of Lenhard and Lenhard [45].

## 3. Results

### 3.1. Evidence of Validity

#### 3.1.1. Based on the Internal Structure

To evaluate the fit of the factorial structures of the risks and resources domains on the FER-R inventory, the scores of 633 participants were taken, with which the CFA-BF was performed, with the 39 risk items and then separately with the 18 resource items. In both cases, unidimensional, oblique, and bifactor models were estimated using the WLSMV estimator to account for the ordinal data [46].

In the risks domain, the unidimensional structure was confirmed with a bifactor model of 39 items, distributed in six facets: (i) *interventions*, 4 items, (ii) *education*, 7 items, (iii) *peers*, 5 items, (iv) *family*, 13 items, (v) *drugs*, 6 items, and (vi) *attitudes*, 4 items. The CFI was = 0.965, the TLI was = 0.961, and the RMSEA was = 0.036 (0.033–0.040).

For the resources domain, the unidimensional structure was confirmed with a bifactor model of 18 items and 2 facets: (vii) *protective resources*, 13 items, and (viii) *the youth’s interests*, 5 items, where the CFI was = 0.978, the TLI was = 0.971, and the RMSEA was = 0.042 (0.035–0.049).

**Figure 3:**
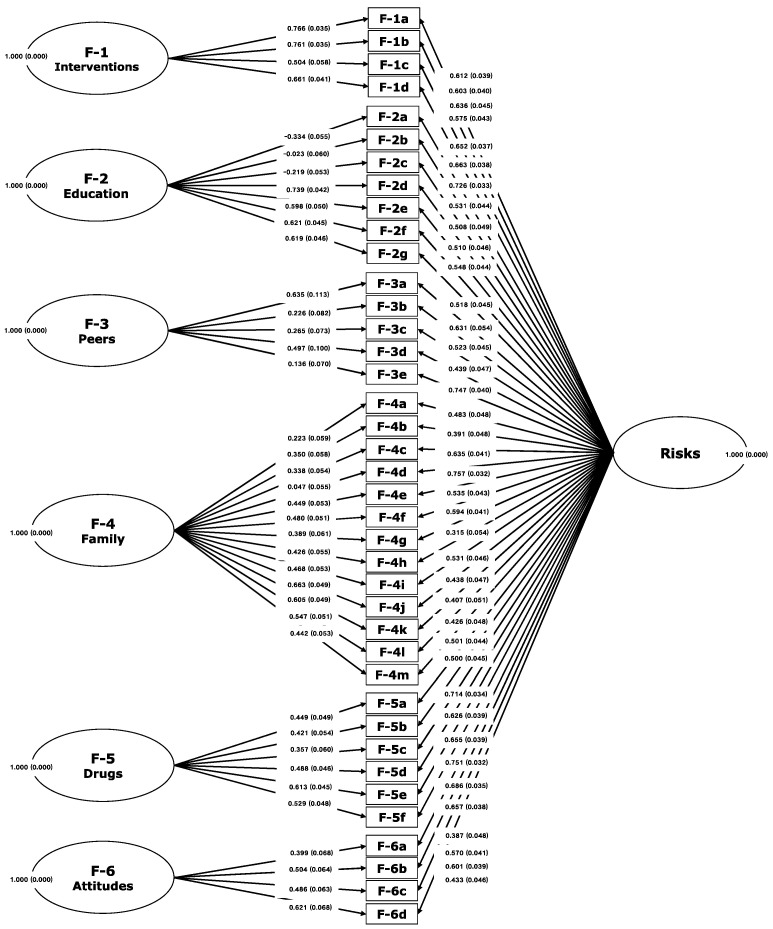
Mapping of confirmatory bifactor analysis of six facets of risks on the FER-R.

**Figure 4:**
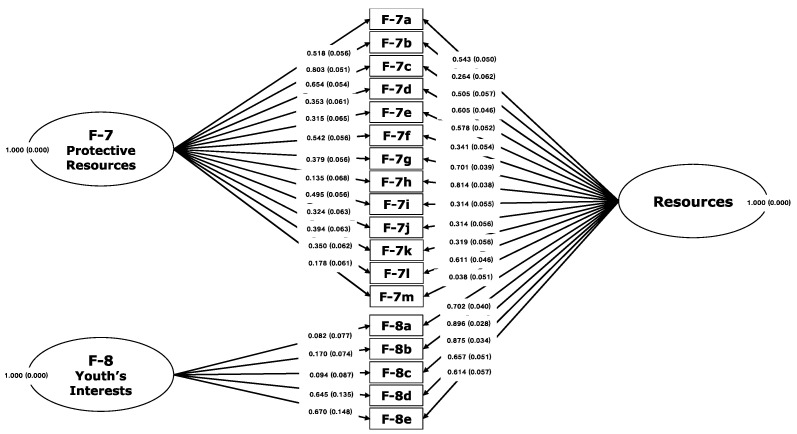
Mapping of confirmatory bifactor analysis of two facets of resources on the FER-R.

As seen in Table 2, the indicators show a good fit between the conceptual framework and the observed data for both models, which is illustrated in Figure 3 and Figure 4. This evidence confirms that the FER-R has a conceptually consistent internal structure with the empirically obtained measurement model.

#### 3.1.2. Based on the Relation to Other Variable: Convergent Validity

The convergent validity of the FER-R was estimated with the *Youth Level Service Case Management Inventory* in its Chilean version, YLS/CMI [17,25]. Using the SPSS 23 program, the two domains and eight facets of the FER-R were correlated with the eight indicators on the YLS/CMI, obtaining values of significant association between the two inventories in the eighty intersections, all with *p* < 0.001. The intersection of total risks on both inventories stands out, with a Rho = 0.919. All the values correlate in a statistically significant way and in the expected direction, i.e., positively between risk factors and negatively between resources on the FER-R and the different risks measured by the YLS/CMI (see Table 3).

The findings reveal a strong association between the variables measured by the two instruments, with moderate-to-high effect sizes. These results are important evidence of the convergent validity of the FER-R when using the most disclosed test in the scientific literature as a criterion [6,13].

#### 3.1.3. Based on the Relation to Other Variables: Divergent Validity

The divergent validity was estimated by correlating the total risk score from the FER-R with two personality-pattern scales from the MACI, not theoretically related to criminogenic risks: (e4) *dramatization* and (e5) *egocentrism*. The values of association in both cases tend to be negative and not statistically significant (*p* > 0.05), which shows the absence of an empirical relation between the evaluated constructs (see Table 4).

#### 3.1.4. Based on the Relation to Other Variables: Discriminant Validity

Discriminant validity was estimated by dividing the sample into two groups according to the criterion of *retrospective criminal recidivism* (presence or absence of judicial antecedents prior to the current sanction) and then comparing the means of both groups in *protective resources*, *youth’s interests*, *and criminogenic risks* using Student’s *t* statistic, and finally estimating the effect size of the observed evidence using Cohen’s d (see Table 5).

### 3.2. Evidence of Reliability

The reliability was obtained with Cronbach’s alpha and Omega Hierarchical (*ωH*), with both being estimated for the two domains. In the risks domain, a *ωH = 0.870* and an *alpha = 0.847* were obtained, and in the resources domain a *ωH = 0.716* and an *alpha = 0.839* were obtained. The results in reliability are satisfactory and can be interpreted unidimensionally, but a deeper analysis of the internal consistency of each facet is required.

### 3.3. Comparative Characterization of the Sample

An exploratory analysis of the scores obtained by males and females in the sample was performed on the risks and resources domains, comparing the medians of each group using the nonparametric Mann–Whitney U test. As noted in Table 6, the results show statistically significant differences in only two of the eight facets that the inventory measures: *interventions* (*p* < 0.05) where the males scored higher than the females, but with a Cohen’s *d* value that means absence of effect; and *the youth’s interests* (*p* < 0.01), where the females scored higher, but with a small effect size.

## 4. Discussion

This study examined the behavior of the FER-R as a risk-and-resources-assessment inventory for a group of 633 Chilean adolescents aged 14 to 19 years, all convicted of crimes. The study of the psychometric properties confirmed that the FER-R is a valid and reliable instrument for the measurement of criminogenic risk factors and protective resources in young offenders (Alarcón et al., 2012).

In relation to the need for evidence of factorial validity for the risk-assessment tools, it was of special interest to recognize the unidimensionality through the bifactor model in the factorial structure of the FER-R for the risks and resources domains. A general factor was determined that combines a static risk facet with five dynamic risk facets was determined in the risks domain. As Mei et al. [10] indicate, examining the construct validity in risk tools is imperative, if in practice they are treated as if they existed without demonstrating it; therefore, this study advances with evidence of internal validity for this construct. Furthermore, the FER-R, being an instrument built in a Latin American context, allows progress in the cultural relevance of these evaluations, especially due to the greater relevance of the family-risk dimension, being able to monitor its factorial structure in different groups and countries of the region if this study is replicated with diverse samples.

In the resources domain, unidimensionality is also confirmed; however, the reliability is slightly lower than in risks. Abbiati et al. [49] indicate that there is little consensus as to how protective factors are operationalized and this may produce less internal consistency, but the FER-R advances the assessment of these factors through its two facets —protective resources and the youth’s interests—with differentiated items, which can act in association with an overall response of adaptation resources.

The reports on instruments that integrate the perspective of risk and resources in their predictive capacity, as described by Viljoen. Et al., [14], are promising in the field of youth justice. Consequently, the evidence of external validity with the YLS/CMI shows a strong association with moderate-to-large effect sizes, both in a positive association for the domain of risks and in a negative one with resources, coinciding with the negative association of risk factors in the YLS/CMI and protective factors of the SAVRY reported by Ortega-Campos et al. (2020). This consolidates a tool that is consistent with the construct of the RNR model. This evidence goes together with the predictive validity reported previously on a sample of 101 adolescent offenders that showed a robust effect size (AUC = 0.73), with a 95% confidence interval (0.63–0.83), comparable to the data reported by Olver et al. [6] and the JAIS system reported by Baird et al. [50].

Finally, it is necessary to highlight that the characterization of the risks and resources of the studied sample did not allow the detection of diversity or differences according to gender or age groups. This can be explained by the limitations of this study, with a higher representation of male adolescents and a very small sample of female adolescents, in addition to a heterogeneous distribution of age groups, given the selectivity of sanction programs, with a higher concentration in adolescents over 16 years of age. To obtain more evidence of validity, it is suggested that the size and statistical power of the sample is increased, follow-up in repeated measures is provided, data in other Latin American countries are collected, and analyses are replicated with more-recent samples, as the data were collected a decade ago. This is a limitation of the study given the far-reaching changes that Chilean society has undergone in recent years that could affect younger generations.

## 5. Conclusions

It can be concluded that the FER-R is a state-of-the-art inventory, based on structured professional judgment, with solid evidence of internal and external validity, which simultaneously includes the evaluation of criminogenic risks and protective factors, and critical aspects considered for assessment instruments of risks (Fazel & Wolf, 2018). From a practical perspective, the determination of the risk profile and the outlining of resources for the intervention are an aid in the targeting of actions according to the criminogenic needs of each case, which facilitates the management of differentiated interventions.

This study is a pioneer given the size and Latin American origin of the sample, and provides evidence that supports the FER-R as a valid instrument for the measurement of criminogenic risk factors and protective resources in Chilean adolescents, the results being encouraging for its international transfer to other Latin American countries. However, it is necessary to increase the size of the sample of females to more accurately observe its functioning with this group. In addition, there is concern about the temporal stability of their structure given the high sensitivity of adolescent behavior to social changes, which requires replicating the study with more-contemporary samples. Finally, it is very important to study the relationship between the variables measured by the FER-R with other variables of the adolescents’ vital context such as emotional regulation, mental health, adverse experiences in childhood and, especially, the presence of criminal organizations in the living environment during an adolescent’s development.

## Figures and Tables

**Table 1:** Characterization of the samples by study, type of sentence, and region of origin.

	Bío Bío		Araucanía		Los Ríos		Los Lagos		Total	
	f	%	f	%	f	%	f	%	f	%
Sampling										
Convicted 2008	730	39%	566	30%	216	11%	374	20%	1.886	100%
Sample obtained	36	5%	120	21%	55	25%	52	14%	263	14%
Convicted 2011–2012	708	38%	605	32%	182	10%	382	20%	1.877	100%
Sample obtained	45	6%	145	24%	126	69%	70	18%	386	21%
Type of Sanction 2008										
Probation	17	47%	74	62%	30	55%	44	85%	165	63%
Imprisonment	19	53%	46	38%	25	45%	8	15%	98	37%
Type of Sanction 2011–2012										
Probation	33	73%	116	80%	86	68%	49	70%	284	74%
Imprisonment	12	27%	29	20%	40	32%	21	30%	102	26%
Total 2008	36	14%	120	46%	55	21%	52	20%	263	100%
Total 2011–2012	45	12%	145	38%	126	33%	70	18%	386	100%

Note: Table prepared by the authors with reference to SENAME data from 2008 to 2013. It is relevant to note that the national territory is divided into 16 regions, with an estimated population of 19.7 million inhabitants in 2021, with the greatest population concentrated in the Metropolitan Region (41.9%). The four study regions indicated in the table are located in the south of Chile, and combined they have 20.3% of the population (https://es.wikipedia.org/wiki/Anexo:Regiones_de_Chile_por_poblaci%C3%B3, accessed on 2 January 2022).

**Table 2:** Indicators of fit of the confirmatory bifactor analysis of the FER-R.

CFA-BF	N	SB-χ^2^	gl	*p*	(SB-χ^2^)/GL	CFI	TLI	RMSEA
Risks 6 Facets	633	1232.221	663	0.000	1.859	0.965	0.961	0.036
Resources 2 Facets	633	249.115	117	0.000	2.129	0.978	0.971	0.042

Note: For the CFI and TLI, an adequate fit of the model was considered when values were higher than 0.90 [47], whereas for the RMSEA, values below 0.08 were considered a reasonable fit [48].

**Table 3:** Convergent validity. Spearman’s Rho correlations between the FER-R and the YLS/CMI.

FER-R	Total Risk YLS/CMI	Offenses	Family Supervision	Education Employment	Relationships with Peers	Drug Addition	Personality Behavior	Attitudes Tendencies
Risks	0.919 **	0.605 **	0.753 **	0.659 **	0.663 **	0.751 **	0.690 **	0.638 **
F1. Interventions	0.587 **	0.838 **	0.415 **	0.290 **	0.427 **	0.451 **	0.369 **	0.310 **
F2. Education	0.725 **	0.391 **	0.521 **	0.763 **	0.470 **	0.529 **	0.538 **	0.469 **
F3. Peers	0.660 **	0.405 **	0.481 **	0.442 **	0.691 **	0.518 **	0.449 **	0.532 **
F4. Family	0.727 **	0.419 **	0.767 **	0.467 **	0.509 **	0.540 **	0.567 **	0.510 **
F5. Drugs	0.747 **	0.465 **	0.534 **	0.510 **	0.527 **	0.863 **	0.525 **	0.463 **
F6. Attitudes	0.672 **	0.310 **	0.522 **	0.483 **	0.422 **	0.399 **	0.670 **	0.698 **
Resources	−0.487 **	−0.204 **	−0.423 **	−0.385 **	−0.360 **	−0.319 **	−0.379 **	−0.537 **
F7. Protective resources	−0.426 **	−0.172 **	−0.400 **	−0.317 **	−0.330 **	−0.271 **	−0.335 **	−0.501 **
F8. Youth’s interests	−0.456 **	−0.197 **	−0.349 **	−0.397 **	−0.314 **	−0.308 **	−0.358 **	−0.444 **

Note: ** Significant correlation at 0.001.

**Table 4:** Divergent validity. Spearman’s Rho correlations between total risks on the FER-R and dramatization and egocentrism on the MACI.

	Risks FER-R		Size Effect
	Rho	*p*	
MACI Dramatization	−0.036	0.447	Null
MACI Egocentrism	−0.053	0.266	Null

**Table 5:** Discriminant validity. Comparison of groups according to retrospective criminal recidivism.

	No History			With Antecedents						
	N	ME	DS	N	ME	DS	*t*	*p*	d	Effect Size
Protective resources	309	8.25	3.40	349	7.37	3.25	−3.391	0.001	0.265	Small
Youth’s Interests	353	3.80	1.64	343	2.44	1.78	−5.889	0.000	0.432	Moderate
Criminogenic risks	357	13.08	9.08	389	22.48	8.02	14.491	0.000	1.099	Large

**Table 6:** Comparative characterization of risks and resources in the FER-R according to the sex of the participants.

	Male (589)		Female (60)							Effect Size
	ME	MD	ME	MD	U	z	p		d^#^	
Risks	16.6	17	14.27	10.5	6868.5	−1.43	0.152	^NS^	----	
F1. Interventions	1.37	1	0.89	0	15,901.0	−2.35	0.019	*	0.159	No effect
F2. Education	2.93	3	2.71	3	16,396.5	−0.82	0.412	^NS^	----	
F3. Peers	3.13	3	3.04	3	17,032.0	−0.30	0.762	^NS^	----	
F4. Family	5.4	5	5.47	4.5	10,551.0	−0.05	0.961	^NS^	----	
F5. Drugs	2.64	3	2.05	1.5	16,983.5	−1.84	0.066	^NS^	----	
F6. Attitudes	1.54	1	1.31	1	16,390.5	−1.28	0.201	^NS^	----	
Resources	9.82	10	10.64	11	11,283.0	−1.28	0.200	^NS^	----	
F7. Protective resources	7.67	8	8.51	9	11,406.0	−1.54	0.123	^NS^	----	
F8. Youth’s interests	2.14	2	3.49	4	14,425.0	−2.90	0.004	**	0.207	Small

Notes: 1. values of statistical significance: ^NS^ = Not significant; * *p* < 0.05; ** *p* < 0.01; 2. d^#^ values estimated with the online calculator from Lenhard and Lenhard [45].

## Data Availability

The data presented in this study are available on request from the corresponding author. The data are not publicly available due to privacy restrictions.
